# Relevant baseline characteristics for describing patients with knee osteoarthritis: results from a Delphi survey

**DOI:** 10.1186/1471-2474-14-369

**Published:** 2013-12-30

**Authors:** Lukas M Wildi, Anita Hensel, Maria Wertli, Beat A Michel, Johann Steurer

**Affiliations:** 1 Department of Rheumatology, University Hospital of Zurich, Gloriastrasse 25, 8091 Zurich, Switzerland; 2 Department of Internal Medicine, Horten Centre for Patient-oriented Research and Knowledge Transfer, University of Zurich, Pestalozzistr, 24, 8091 Zurich, Switzerland

**Keywords:** Knee osteoarthritis, Baseline characteristics, Inclusion criteria, Comparability, Applicability, Clinical trial

## Abstract

**Background:**

Inclusion/exclusion criteria and baseline characteristics are essential for assessing the applicability of trial results to a given patient and the comparability of study populations for meta-analyses. This Delphi survey aimed to generate a set of baseline characteristics for describing patients with knee osteoarthritis enrolled in clinical studies.

**Methods:**

Survey participants comprised clinical experts (n = 23; mean age 54 y; from 4 continents) that had authored at least two randomized trials on knee osteoarthritis. First, given a prepared list of baseline patient characteristics, the experts were asked to add characteristics they considered important for assessing comparability of patient populations in different trials that evaluated the efficacy of non-surgical interventions for treating knee osteoarthritis. Next, they were asked to rate the importance of each characteristic, on a scale of 0 (not important) to 10 (highly important), according to three outcome categories: pain, function, and structure.

**Results:**

Participants identified 121 baseline characteristics. A rating ≥7 points was assigned to 39 characteristics (e.g., age, depression, global knee pain, daily dose of pain killers, Kellgren-Lawrence grading); of these, 20 were related to pain, 15 to function, and 23 to structural outcomes. Global knee pain was the only baseline characteristic that fulfilled among experts the predefined consensus criteria.

**Conclusions:**

Experts identified a large number of characteristics for describing patients with knee osteoarthritis. Disagreement and uncertainty prevailed over the relevance of these characteristics. Our findings justified further efforts to define appropriate, broadly acceptable sets of baseline characteristics for describing patients with knee osteoarthritis.

## Background

After reading and critically appraising a publication on the effects of a particular treatment, clinicians must consider the patients to which the reported results might apply. The study authors typically present inclusion/exclusion criteria in the Methods section and the baseline characteristics of the included patients in the Results section (typically in Table 1). Inclusion/exclusion criteria inform readers how eligible patients were selected (e.g., age, illness, duration of complaints, severity of illness, and co-morbidities) for participating in the trial. Baseline characteristics describe the participants within a given boundary of inclusion/exclusion criteria. The reported baseline characteristics represent, in general, prognostic factors that can impact the future course of the illness. For example, among patients with knee osteoarthritis, those with knee malalignments have a less favorable future course than patients without malalignments [1].

**Table 1:** List of relevant baseline characteristics

	**Outcome**		
	**Pain**	**Function**	**Structure**
*General information*			
	Age	Age	Age
	Gender	Gender	Gender
	Activity level in daily life	Activity level in daily life	
*Psychosocial factors*			
	Pain catastrophizing		
	Anxiety and Fear		
	Depression	Depression	
	Quality of life*	Quality of life*	
*Information from history*			
			Primary or secondary osteoarthritis
	Duration since onset of symptoms indicating knee osteoarthritis	Duration since onset of symptoms indicating knee osteoarthritis	Duration since onset of symptoms indicating knee osteoarthritis
	Intermittent or constant pain	Intermittent or constant pain	
	Pain remained constant or varied in the last week		
	Average daily pain during the last week	Average daily pain during the last week	
		Walking aid needed (e.g., cane/walker)	
	Global knee pain**	Global knee pain**	Global knee pain**
	Waking up with pain		
	Weight bearing pain	Weight bearing pain	
	Pain at rest		
	Pain when going down stairs		
	Function of knee***	Function of knee***	Function of knee***
	Pain or reduced function in other joints (hip/spine)	Pain or reduced function in other joints (hip/spine)	
			Previous history of knee trauma
			Previous history of meniscal tear or meniscectomy
			Previous history of ACL^§^ rupture
			Previous surgical intervention
	Daily dose of pain killers (NSAIDs)		
*Information from physical examination*			
	BMI	BMI	BMI
		Physical functional ability; e.g., performance tests of baseline physical ability (e.g., the Timed Up and Go test or a 6-min walking test)	
*Lab results*			
*Imaging results*			
			Kellgren-Lawrence grading (weight bearing)
			Bone marrow lesions
			Joint space width
			Synovitis (US^§§^, MRI^§§§^)
			Effusion (US, MRI)
			Involved joint compartment (i.e., patellofemoral vs. tibiofemoral vs. combined etc.)
			Meniscus tear
			Meniscus extrusion
			ACL tear
			Cartilage abnormalities (MRI)
			Malalignment (hip-knee-ankle mechanical axis)
			Cartilage volume/thickness in the compartment of interest (MRI)

Legend: List of baseline characteristics with a median rating of importance ≥7 according to three different outcome categories, based on the opinions of an expert panel.

*Abbreviations:* *e.g., SF-36; **e.g., VAS, WOMAC; ***e.g., WOMAC, Lequesne; § Anterior crucial ligament; §§ Ultrasound; §§§ Magnetic Resonance Imaging.

Detailed baseline characteristics of trial participants are also important for researchers in conducting systematic reviews and meta-analyses. Guidelines for preparing meta-analyses and systematic reviews recommend assessing the comparability of patient populations in different primary studies and determining whether it is reasonable to combine the results in a single value [2-4]. An appropriate comparison is possible only when the required data are reported in primary studies. Reported baseline characteristics may be incomplete. Improper attention to the comparability between patients of different trials may raise criticism of results. For example, a systematic review that compared the effects of chondroitin or glucosamine in patients with knee osteoarthritis should ensure the patient populations were comparable [5]. To our knowledge, recommendations from experts are lacking on selecting the most relevant baseline characteristics for patients with osteoarthritis of the knee.

The aim of this survey was to generate a set of baseline characteristics that could, based on expert opinions, appropriately describe patients with knee osteoarthritis enrolled in clinical studies.

## Methods

We assembled an international panel of clinical experts on osteoarthritis to generate a list of baseline characteristics for describing patients included in clinical trials with knee osteoarthritis. A preliminary list was prepared and sent to these experts with the request that they add patient characteristics that they considered relevant. In a second round, the experts were asked to rate the relevance of each patient characteristic.

### Selection and recruitment of experts

We searched Medline and EMBASE to identify clinical experts on osteoarthritis of the knee. The following MESH terms were used: Osteoarthritis, Knee, Physical Therapy Modalities, Steroids, Viscosupplementation, Anti-Inflammatory Agents, Non-Steroidal, Randomized controlled trial (a detailed list of the search strategy is available upon request from the corresponding author). The search was restricted to articles published between the years 2007 and 2012. We included only studies that evaluated the treatment effect of steroid injections, viscosupplementation, non-steroidal analgesics, or physical therapy. The aim, set arbitrarily, was to identify 20 experts that would participate in our survey. From the list of all authors, we selected those that co-authored three or more trials plus a random sample of authors that were listed on two publications. Based on the medical specialty and/or affiliation mentioned in the publication, we categorized authors into groups of clinically-oriented (e.g., rheumatologists, physiotherapists) or methodology-oriented (e.g., clinical epidemiologists, biostatisticians) researchers. Only authors categorized as clinically-oriented researchers were contacted for participation in the survey.

### First round

The selected experts were contacted by E-mail and informed about the aim of the study. Those that agreed to participate received a prepared form and a request to add characteristics to complete a preliminary list of baseline characteristics (indicated by § in Additional file 1).

The experts received the following information: Patients with knee osteoarthritis have been included in four randomized trials (A/B/C/D, Additional file 2). In each of the four trials, one group of patients received an active treatment X (non-surgical) and the other group a placebo. In trials A and B, the outcome of interest was pain; in trials C and D, the outcome was a functional parameter. The results between trials differed significantly. In trials A and C, treatment X showed a significant benefit, and in trials B and D, the identical treatment X showed no benefit. The execution of the trials (intervention, treatment, measurement of outcomes, randomization etc.) was identical; however, one reason for the contradictory results may have been the inclusion of different patient populations in all four trials. Then, the experts were asked “From your experience as a clinical expert, what baseline patient characteristics would be necessary to identify patients to whom the trial results might be applicable, and furthermore, to evaluate the comparability of the two populations in trials A/B and C/D? The participants were asked to add characteristics that they thought should be included in an attached list of baseline characteristics. Participants returned the completed lists by mail or fax.

### Second round

Based on the answers from the first round of inquiries, we updated the list of patient characteristics. To avoid redundancy, nearly identical characteristics were merged into one parameter. The final list included 121 items that were assigned to one of six categories, including general information about the patient (e.g., age, gender), psychosocial factors (e.g., depression, anxiety), history (e.g., duration of pain, pain provoking maneuvers), physical examination results (e.g., periarticular tenderness, instability), laboratory tests (e.g., C-reactive protein, serum hyaluronic acid concentration), and imaging results (e.g., Kellgren-Lawrence grading, bone marrow lesion).

This updated list was sent to the experts that had returned the questionnaire from the first round. The participants were asked to rate the ‘importance’ of each baseline characteristic on a scale of 0 to 10, where 0 indicated no importance, and 10 indicated the utmost importance. We informed participants that the ‘degree of importance’ was related to two issues; first, the relevance of the characteristic in identifying patients in daily practice to whom the results of a study were applicable; and, second, its usefulness in meta-analyses for assessing the comparability of patient populations from different primary trials for a potential pooling of results.

We reasoned that the experts would probably estimate the importance of baseline characteristics differently, depending on the outcome of interest. Therefore, we asked the participants to rate each baseline characteristic according to three different outcome categories; pain (e.g., VAS), function (e.g., WOMAC-function sub-score), and structure (e.g., change of the joint space width over time).

Participants that completed both questionnaires were asked to provide information about their age, gender, and primary medical specialty. Completed questionnaires were returned by E-mail or fax. All experts that responded in the first round were informed that they would receive a voucher for $100 after returning the completed questionnaire for round 2.

### Statistical analysis

For this study, the medians and interquartile ranges for each parameter were calculated to quantify the importance assigned to single items. The median is a measure for the average and the 25-75% interquartile range (IQR) and the range are measures of dispersion of values. The median value means that half of the values are below and half the values above the median value. The 25-75% IQR is the difference between the values of the 25th and 75th percentiles. The 0th and 100th percentiles (minimal and maximal values) define the range. The final list only included baseline characteristics with a median rating ≥7 (on a 0 to 10 point scale). We arbitrarily defined a consensus among experts as a rating with an interquartile range ≤4 (±2) points.

### Ethical approval

This work did not involve human subjects or animals. Thus, according to national laws and institutional regulations, review board (IRB) approval was not necessary. All experts acknowledged in the manuscript gave their permission to list their names.

## Results

### Recruitment and selection of experts

Between 2007 and 2012, we identified 364 randomized trials involving patients with osteoarthritis of the knee. From 1149 authors, 76 were invited to participate in the survey. Of those, 32 agreed to participate, and 23 finally took part in both rounds. The mean age of the panel members was 54 years (range 39 to 75 years); 7 of the participants were female. Medical specialties of the panel members included rheumatologists (n = 13), physiotherapy (n = 6), rehabilitation medicine (n = 1), occupational therapy (n = 1), orthopedic biomechanics (n = 1), and musculoskeletal medicine (n = 1). Nine panel members were from North America (USA/Canada), eleven from Europe, two from Australia, and one from Asia.

### Results of first round

Twenty-three of 32 experts returned the list with additional baseline characteristics. The additions comprised a total of 267 baseline characteristics in addition to the 30 characteristics nominated in the first list sent out. After deleting repeated characteristics and merging highly similar characteristics, 121 remained in the final list (Additional file 1).

### Results of second round

All 23 participants from the first round completed the questionnaire in round 2. The medians of the expert ratings varied between 1 (e.g., click on knee motion) and 10 (e.g., global knee pain, WOMAC score).

To ensure the list of baseline characteristics remained within reasonable limits, we only included characteristics that had been rated a median of seven or above by the expert panel. A total of 39 characteristics fulfilled this criterion; 20 were related to a pain-reduction outcome, 15 were related to a functional improvement outcome, and 23 to a structural improvement outcome. The list of baseline characteristics with a median rating ≥7 is displayed in Table 1. Details of the median, 25-75% interquartile range, and the range of estimates are shown in Additional file 1. Six baseline characteristics were rated ≥7 in all three outcome categories (age, gender, BMI, global knee pain, function of knee, duration since onset of symptoms indicating knee osteoarthritis).

A consensus on the relevance of a baseline characteristic was arbitrarily defined as a calculated range of four (± 2) points or less around the median. Only one parameter, the global knee pain (e.g., VAS, WOMAC), fulfilled this criterion; all other characteristics listed in Table 1 displayed ranges greater than four points but are still rated as relevant baseline characteristics.

## Discussion

There were three main results of this study. First, experts listed a large number of baseline characteristics that described patients with osteoarthritis of the knee included in trials that evaluated treatment effects. Second, experts agreed on the relevance of only one baseline characteristic. All other baseline characteristics received ratings scattered over a broad range, which indicated disagreement among experts. Third, the relevance of baseline characteristics varied according to the outcome measure in a trial.

Researchers have published a number of relevant articles that emphasized the definitions and measurements of outcomes in clinical trials that evaluated treatment effects in patients with knee osteoarthritis [6-9]. Despite a thorough search in various databases, we could not find any publications that focused on how to select baseline characteristics of patients that participated in trials on osteoarthritis of the knee. However, we identified a few publications that summarized the evidence for prognostic factors that characterized patients with knee osteoarthritis. Cheung et al. [1] stated that strong or moderate evidence indicated that progression was associated with age, generalized osteoarthritis, knee malalignment, and serum hyaluronic acid concentration; limited evidence indicated associations with knee pain, synovitis, the adduction moment of the knee, vitamin D and C concentrations, and MRI bone marrow lesions in the knee; and conflicting evidence indicated associations with body mass index, initial severity of x-ray changes, cartilage oligomeric protein (Comp), and urinary CTX-II. In a recent systematic review, Chapple et al. [10] reported some of the same results. They found that age, generalized osteoarthritis, varus knee alignment, and radiographic features, particularly joint space narrowing were strongly associated with prognosis. The latter review [10] provided no specific statements about the prognostic relevance of serum hyaluronic acid concentration.

In part, our results were in agreement with the previous studies [1,10]; but in part, our findings disagreed with those studies. For example, the panel members of our survey considered psychosocial factors important, e.g., anxiety and fear; however, the supporting evidence for these factors appeared to be scarce. The most striking discrepancy was the difference between the number of prognostic factors gathered from the synthesis of original studies and the number collected from the clinical experts of the present study. The clinical experts listed a much higher number of relevant factors than the numbers listed in the current literature.

The results of our survey might be helpful for clinicians and researchers. This study aimed to provide guidance to clinicians for assessing the applicability of trial results to a different clinical application. After reading the results of a clinical trial, the main task of the clinician is to assess which patients might benefit from the treatment. Apart from the inclusion/exclusion criteria, the most significant information for this assessment are the baseline characteristics of study participants. The present study provides a list of relevant factors based on clinical expert opinions. Clinicians can consult this list to evaluate the comprehensiveness of the baseline characteristics in the reports they are considering.

Researchers may also find this list of baseline characteristics important for two reasons. First, our results may inform the design of future trials in patients with knee osteoarthritis. Researchers can consult the present list of baseline characteristics for each outcome of interest to decide which patient characteristics should be reported. The careful selection and reporting of baseline characteristics can facilitate the translation of research results into patient care, and this increases the usefulness of trial results. Second, researchers may find the list relevant when synthesizing the results of original studies. Guidelines for preparing systematic reviews by meta-analyses recommend checking the comparability of patient populations between original studies before pooling the results to derive a single value [2,4,11]. A prerequisite for this type of assessment is the availability of detailed information about the distribution of baseline characteristics among the patients included in the original studies.

Our study had both strengths and limitations. The primary limitation, inherent in most surveys, was that a different panel of experts may provide different results. A strength was that the members of the panel were experts in the field and had authored two or more clinical trials that evaluated the effects of treatments for patients with osteoarthritis of the knee. Furthermore, we included a large number of panel members, and they were from different countries. A panel with about 15 members is recommended for surveys to reach a consensus or to assess the degree of disconsensus [12]. With 23 panelists, we exceeded that recommended number. The panel member internationality assured a broad spectrum of opinions and eliminated the domination of an opinion based on a single clinic or region-specific beliefs. A further limitation of our study was that, in the first questionnaire, we only included pain-related and functional outcomes, but no structural outcomes. In the second questionnaire, we included the structural outcomes. We assume that the addition of an outcome parameter did not impact the results different results.

## Conclusions

In conclusion, it remains uncertain which baseline characteristics are most important to collect and report in knee osteoarthritis trials. We cannot claim that our results provided a standard for reporting baseline characteristics. However, the results of this survey may serve to guide clinicians and meta-analysts in assessing whether the baseline characteristics of a given clinical trial are comprehensively reported. In addition, we provided a list of characteristics considered important for the respective study outcomes, based on the opinions of an expert panel. Finally, the extent of disagreement among experts on the relevance of baseline characteristics should motivate further research.

### Participating experts (alphabetic order)

R. Altman, Los Angeles, USA; M. Akai, Saitama, Japan; K. L. Bennell, Melbourne, Australia; F. Berenbaum, Paris, France; X. Chevalier, Paris, France; A. Fioravanti, Siena, Italy; G. K. Fitzgerald, Pittsburgh, USA; N. Foster, Staffordshire, UK; B. Haraoui, Montreal, Canada; M. Henriksen, Frederiksberg, Denmark; G. Herrero-Beaumont, Madrid, Spain; R. S. Hinman, Melbourne, Australien; H. Lund, Odense, Denmark; E. Maheu, Paris, France; J. Martel-Pelletier, Montreal, Canada; R. W. Moskowitz, Cleveland, USA; S. L. Murphy, Michigan, USA; K. Pavelka, Prag, Tschechien; JP. Pelletier, Montreal, Canada; JP. Raynauld, Montreal, Canada; D. Rosenbaum, Münster, Germany; T. J. Schnitzer, Chicago, USA; N. E. Walsh, Bristol, England.

## Competing interests

We declare no competing interests. This study was funded by the Horten Center and University Hospital of Zurich. The funding bodies had no influence on the design of the study, the analysis of data and the writing of the manuscript.

## Authors’ contributions

All authors made substantial contributions to the analysis and interpretation of data, the critical manuscript revision, and the final manuscript approval. LMW, AH, BAM, and JS were responsible for the conception and the design of the study, and they drafted the manuscript. AH collected and assembled the data.

## Pre-publication history

The pre-publication history for this paper can be accessed here:

http://www.biomedcentral.com/1471-2474/14/369/prepub

## Supplementary Material

Additional file 1 Complete list of baseline characteristics and ratings of the importance. Click here for file

Additional file 2 Interventions, outcome measures, and results for four fictitious trials presented to the expert panel. Click here for file
