# Supplementary material for: Relevant baseline characteristics for describing patients with knee osteoarthritis: results from a Delphi survey
Source: BMC Musculoskelet Disord. 2013 Dec 30;14:369. doi: 10.1186/1471-2474-14-369 (PMC3882493; doi:10.1186/1471-2474-14-369)
Supplement: Additional file 1 — Complete list of baseline characteristics and ratings of the importance. [file 1471-2474-14-369-S1.docx]

**Appendix 1:** Complete list of baseline characteristics and ratings of the importance

|  | **Pain** | | | **Function** | | | **Structure** | | |
| --- | --- | --- | --- | --- | --- | --- | --- | --- | --- |
| **Characteristics** | **Median** | **25-75%  IQR** | **Range** | **Median** | **25-75% IQR** | **Range** | **Median** | **25-75% IQR** | **Range** |
| Age^§^ | 8 | 5-10 | 0-10 | 10 | 8-10 | 2-10 | 10 | 6.5-10 | 0-10 |
| Gender^§^ | 8 | 5-10 | 1-10 | 8 | 4.5-10 | 0-10 | 8 | 2.5-10 | 0-10 |
| Ethnicity | 5 | 2.5-7.5 | 0-10 | 5 | 2.5-7.5 | 0-10 | 5 | 0.5-7.5 | 0-10 |
| Occupation/Workplace exposure | 6 | 5-7 | 3-10 | 6 | 5.5-7 | 2-10 | 6 | 4-8 | 0-10 |
| Employment status | 4 | 2-6 | 0-10 | 4 | 1-6.5 | 0-10 | 3 | 0.5-5 | 0-10 |
| Education level | 5 | 3-6 | 0-10 | 4 | 3-6 | 0-7 | 3 | 0-4 | 0-6 |
| Marital status | 2 | 0-4 | 0-6 | 2 | 0-4.5 | 0-6 | 0 | 0-2 | 0-6 |
| Activity level in daily life | 7 | 4.5-8 | 3-10 | 7 | 5-8 | 2-10 | 6 | 4-8 | 0-10 |
| Sports Participation | 5 | 3.5-6.5 | 0-10 | 5 | 3.5-7 | 0-10 | 5 | 5-7.5 | 0-10 |
| Disambiguation /dominant leg (right or left leg for kicking a ball) | 1 | 0-3.5 | 0-10 | 2 | 0-4.5 | 0-10 | 1 | 0-5 | 0-10 |
| If female: menopause and age of menopause | 2 | 0-5 | 0-7 | 3 | 0.5-5.5 | 0-8 | 4 | 3-7 | 0-10 |
| Setting from which the participants were recruited (e.g., primary care) | 5 | 3-7 | 0-10 | 5 | 3-7 | 0-10 | 5 | 2-7 | 0-10 |
|  |  |  |  |  |  |  |  |  |  |
| Self-efficacy | 6 | 3-7 | 0-10 | 5 | 3-7 | 0-10 | 0 | 0-3.5 | 0-8 |
| Pain catastrophizing | 8 | 6-8.5 | 0-10 | 5 | 3.5-8 | 0-9 | 0 | 0-2.5 | 0-7 |
| Anxiety and Fear | 8 | 6-8 | 0-10 | 6 | 5-7.5 | 0-10 | 0 | 0-2.5 | 0-7 |
| Depression | 7 | 6.5-8 | 2-10 | 7 | 5-8 | 2-10 | 0 | 0-3 | 0-8 |
| Readiness to change behavior | 6 | 4-8 | 0-10 | 6 | 3.5-8 | 0-9 | 0 | 0-1 | 0-9 |
| Health beliefs | 5 | 3.5-7 | 0-10 | 5 | 3-7 | 0-9 | 0 | 0-0 | 0-7 |
| Perceptions about the knee problem and its treatment (e.g., illness perceptions) | 5 | 5-8 | 0-10 | 5 | 4-7.5 | 0-9 | 0 | 0-1 | 0-9 |
| Expectations about what the future holds (outcome expectations) | 6 | 5-7.5 | 2-10 | 5 | 5-7 | 1-9 | 0 | 0-0 | 0-6 |
| Quality of life (e.g., SF-36) | 7 | 6-8 | 3-10 | 8 | 6-8.5 | 3-10 | 2 | 0-5 | 0-9 |
|  |  |  |  |  |  |  |  |  |  |
| Classification of osteoarthritis (primary or secondary) | 3 | 0-8 | 0-10 | 4 | 0.5-8 | 0-10 | 8 | 5-9 | 0-10 |
| Duration since onset of symptoms indicating knee osteoarthritis^§^ | 7 | 5-8 | 1-10 | 7 | 6-8 | 1-10 | 7 | 5-8 | 0-10 |
| Intermittent pain or constant pain | 8 | 6-10 | 2-10 | 7 | 5.5-8 | 0-10 | 4 | 0.5-7.5 | 0-10 |
| Global spontaneous pain during the previous 48 hours | 6 | 2-10 | 0-10 | 5 | 2.5-8 | 0-10 | 1 | 0-6 | 0-10 |
| Pain remained constant or varied in the last week | 7 | 4-9 | 0-10 | 5 | 2.5-8 | 0-10 | 2 | 0-5.5 | 0-10 |
| Average daily pain during the last week | 8 | 7-10 | 2-10 | 8 | 6-8.5 | 0-10 | 4 | 0-7.5 | 0-10 |
| Localization of pain: medial/lateral periarticular, anterior/posterior periarticular, intra-articular^§^ | 5 | 2-7.5 | 0-10 | 3 | 1.5-6.5 | 0-10 | 2 | 0-6 | 0-10 |
| Unilateral or bilateral presentation | 5 | 3.5-8 | 0-10 | 6 | 4.5-8 | 0-10 | 5 | 1-7.5 | 0-10 |
| Episodes of knee swelling/effusion (disease flares) | 6 | 4.5-8 | 0-10 | 6 | 5-8.5 | 0-10 | 6 | 4.5-7.5 | 0-10 |
| Self-reported instability of knee | 4 | 2.5-6 | 0-10 | 5 | 2-7.5 | 0-10 | 4 | 2-6 | 0-10 |
| Walking aid needed (e.g. cane/walker) | 6 | 3-8 | 0-10 | 8 | 5.5-9 | 0-10 | 4 | 1-6 | 0-10 |
| Global knee pain (e.g. VAS, WOMAC) | 10 | 8-10 | 6-10 | 9 | 6.5-10 | 3-10 | 8 | 4-10 | 0-10 |
| Pain during sleep - waking up with pain (inflammatory pain) | 8 | 5.5-10 | 2-10 | 6 | 5-9 | 0-10 | 5 | 3.5-8 | 0-10 |
| Weight bearing pain (mechanical pain) | 8 | 7-10 | 0-10 | 7 | 5-8 | 0-10 | 6 | 2-9 | 0-10 |
| Tingling and/or burning pain (neuropathic pain) | 5 | 4-8 | 0-10 | 5 | 2.5-6.5 | 0-10 | 0 | 0-4.5 | 0-10 |
| Pain at rest^§^ | 8 | 4.5-9 | 0-10 | 5 | 2-7.5 | 0-10 | 3 | 0-7 | 0-10 |
| Pain when walking downhill^§^ | 6 | 4-8 | 0-10 | 6 | 2.5-7.5 | 0-10 | 2 | 0-6 | 0-10 |
| Pain when going down stairs | 7 | 4-8 | 0-10 | 6 | 4.5-8 | 0-10 | 3 | 0-7 | 0-10 |
| Pain when walking on a level surface | 6 | 5-8.5 | 0-10 | 6 | 5-7.5 | 0-10 | 4 | 0-6 | 0-10 |
| Pain after exercise | 6 | 3.5-8 | 0-10 | 5 | 4-8 | 0-10 | 3 | 0-5.5 | 0-8 |
| Pain on starting an action (e.g., walking) | 6 | 4-8 | 0-10 | 5 | 3.5-7 | 0-10 | 3 | 0-5.5 | 0-10 |
| Function of knee (e.g., WOMAC, Lequesne) | 8 | 5-10 | 3-10 | 10 | 8-10 | 3-10 | 8 | 5.5-10 | 0-10 |
| Stiffness of knee (e.g., WOMAC) | 5 | 3-6 | 0-10 | 6 | 3.5-8 | 0-10 | 4 | 0-5.5 | 0-10 |
| Morning stiffness | 4 | 3-6.5 | 0-10 | 4 | 3-7 | 0-10 | 3 | 0-5 | 0-10 |
| Vascular or neurological claudication | 3 | 0-5.5 | 0-10 | 3 | 0-6 | 0-10 | 0 | 0-2 | 0-7 |
| Widespread pain (according to new criteria for fibromyalgia) | 6 | 3-8 | 0-10 | 5 | 2.5-8 | 0-10 | 0 | 0-3 | 0-8 |
| Pain/reduced function in other joints (hip/spine) | 7 | 3-8 | 0-10 | 7 | 3-8 | 0-10 | 1 | 0-3.5 | 0-10 |
| Comorbidities^§^ | 6 | 4-8 | 0-10 | 6 | 5-8.5 | 0-10 | 3 | 0-5.5 | 0-10 |
| Burden of somatic symptoms (joint and other) | 5 | 0-7 | 0-10 | 4 | 0-7 | 0-10 | 0 | 0-2.75 | 0-8 |
| Clinical phenotypes (e.g., genetic, estrogen hormone dependent, age related) | 2 | 0-4.75 | 0-10 | 2 | 0.25-5.75 | 0-10 | 2 | 0-5 | 0-10 |
| Previous history of trauma to the knee | 5 | 2-7.5 | 0-10 | 5 | 2-7.5 | 0-10 | 8 | 5.5-9 | 0-10 |
| Previous history of meniscal tear or menisectomy | 4 | 2-8 | 0-10 | 5 | 2-7.5 | 0-10 | 8 | 7.5-10 | 0-10 |
| Previous history of ACL rupture | 3 | 2-8 | 0-10 | 5 | 2-7.5 | 0-10 | 8 | 7.5-10 | 0-10 |
| Previous surgical intervention | 5 | 2-8 | 0-10 | 5 | 2.5-8 | 0-10 | 8 | 8-10 | 4-10 |
| Previous treatment with opioids | 4 | 0.5-8 | 0-10 | 4 | 0-6 | 0-10 | 0 | 0-1.5 | 0-8 |
| Previous treatment with paracetamol | 4 | 0-6.5 | 0-10 | 4 | 0-6.5 | 0-10 | 0 | 0-2 | 0-8 |
| Previous treatment with intra-articular steroids | 4 | 1.5-7.5 | 0-10 | 4 | 1-5.5 | 0-10 | 1 | 0-4.5 | 0-10 |
| Previous treatment with NSAIDs | 4 | 2.5-8 | 0-10 | 4 | 2.5-7.5 | 0-10 | 1 | 0-4 | 0-10 |
| Previous treatment with i.a. viscosupplementation | 5 | 2.5-7 | 0-10 | 4 | 2-6 | 0-10 | 2 | 0-5 | 0-10 |
| Previous physical (exercise) therapy | 3 | 1.5-5.5 | 0-10 | 3 | 1.5-5.5 | 0-10 | 2 | 0-3 | 0-10 |
| Daily dose of pain killers (NSAIDs) | 7 | 4.5-8 | 0-10 | 6 | 4-8 | 0-10 | 1 | 0-7 | 0-10 |
| Family history of knee osteoarthritis | 4 | 1-5 | 0-10 | 3 | 1.5-5 | 0-9 | 3 | 0.5-5 | 0-10 |
|  |  |  |  |  |  |  |  |  |  |
| Body Mass Index(BMI)^§^ | 8 | 8-10 | 2-10 | 8 | 8-10 | 2-10 | 9 | 7-10 | 2-10 |
| Hip-to-waist ratio | 2 | 0-4 | 0-10 | 2 | 0-4 | 0-10 | 0.5 | 0-3 | 0-10 |
| Waist circumference | 3.5 | 0-5 | 0-10 | 3.5 | 0-5 | 0-10 | 3.5 | 0-6 | 0-10 |
| Periarticular tenderness over the tibial collateral ligament^§^ | 2 | 0.25-4.5 | 0-10 | 1 | 0-3 | 0-10 | 0 | 0-1 | 0-6 |
| Periarticular tenderness over the fibular collateral ligament^§^ | 1.5 | 0-4.5 | 0-10 | 1 | 0-3 | 0-10 | 0 | 0-1 | 0-6 |
| Periarticular tenderness at the *pes* *anserinus*^§^ | 1.5 | 0-5 | 0-10 | 1 | 0-3.75 | 0-10 | 0 | 0-1 | 0-6 |
| Tenderness of the medial joint space line^§^ | 2.5 | 0.25-4.75 | 0-10 | 2 | 0.25-3.75 | 0-10 | 0 | 0-3.75 | 0-9 |
| Tenderness of the lateral joint space line^§^ | 2 | 0-4 | 0-10 | 2 | 0-3 | 0-10 | 0 | 0-2.75 | 0-9 |
| Muscle tenderness | 1 | 0-4.75 | 0-10 | 0.5 | 0-5.5 | 0-10 | 0 | 0-0.75 | 0-9 |
| Anterior instability^§^ | 3.5 | 0.25-5 | 0-10 | 4.5 | 1.25-6.75 | 0-10 | 2.5 | 0.5-5.75 | 0-10 |
| Posterior instability^§^ | 3 | 0-5 | 0-10 | 4 | 0.25-6.75 | 0-10 | 2 | 0-5.75 | 0-10 |
| Medial instability^§^ | 3.5 | 1-5 | 0-10 | 5 | 1.25-6.75 | 0-10 | 3 | 0.5-6 | 0-10 |
| Lateral instability^§^ | 3 | 0.25-5 | 0-10 | 4 | 1-6 | 0-10 | 3 | 0-6 | 0-10 |
| Joint effusion (indicated with a ballottement of the patella)^§^ | 5 | 2.5-8 | 0-10 | 5 | 2-8 | 0-10 | 4.5 | 2-7 | 0-10 |
| Joint swelling | 6 | 1.25-7.75 | 0-10 | 5 | 1.25-7.75 | 0-10 | 5 | 0-7 | 0-10 |
| Increased skin temperature in the knee region | 1 | 0-6.5 | 0-10 | 1 | 0-3 | 0-10 | 1 | 0-5.25 | 0-9 |
| Quadriceps strength | 4.5 | 1.25-5 | 0-10 | 5 | 2.25-8 | 0-10 | 2.5 | 2-4.75 | 0-9 |
| Knee extensor/flexor strength ratio | 1.5 | 0-2 | 0-10 | 2.5 | 0-5 | 0-10 | 0 | 0-2 | 0-9 |
| Alignment of the lower limbs | 2 | 0-5 | 0-10 | 2 | 0-5 | 0-10 | 2 | 0-6.75 | 0-10 |
| Leg length discrepancy | 2 | 0-4.75 | 0-10 | 2 | 0-5 | 0-10 | 2.5 | 0-5 | 0-9 |
| Knee adduction moment (gait analysis) | 2 | 0-4.5 | 0-10 | 2 | 0-5 | 0-10 | 2.5 | 0-6 | 0-9 |
| Knee proprioception | 1.5 | 0-4.75 | 0-10 | 1.5 | 0-4 | 0-10 | 0.5 | 0-3 | 0-6 |
| Pain and/or crepitation, indicated by pressing or rocking the patella | 2.5 | 0-4.75 | 0-10 | 2 | 0-3.75 | 0-10 | 0 | 0-2.75 | 0-5 |
| Crepitation on knee motion | 2.5 | 0-5 | 0-10 | 2.5 | 0-4 | 0-8 | 0 | 0-4 | 0-8 |
| Click on knee motion | 1 | 0-3 | 0-10 | 1 | 0-3 | 0-5 | 0 | 0-3.75 | 0-5 |
| Range of motion (knee) | 2.5 | 0.25-4.5 | 0-10 | 5 | 2-7.75 | 0-10 | 3.5 | 0-5 | 0-10 |
| Range of motion (hip) | 2.5 | 0-5 | 0-10 | 4.5 | 2.5-5 | 0-9 | 0 | 0-3 | 0-8 |
| Pain on terminal flexion/extension of knee | 1.5 | 0-4.75 | 0-10 | 2 | 0-4.75 | 0-10 | 0 | 0-2 | 0-9 |
| Passive or active stiffness | 1.5 | 0-3.75 | 0-10 | 2 | 0-4.75 | 0-8 | 0 | 0-2 | 0-8 |
| Palpable osteophytes | 2 | 0-3.75 | 0-10 | 2 | 0-3 | 0-8 | 2 | 0-4.75 | 0-10 |
| Circumference of the thigh (10 cm above superior patellar pole) | 0 | 0-2 | 0-10 | 1.5 | 0-3.75 | 0-9 | 0 | 0-1.5 | 0-4 |
| Meniscus provocation tests | 1.5 | 0-4.75 | 0-10 | 1 | 0-3.75 | 0-10 | 2 | 0-4.75 | 0-6 |
| Physical functional ability (e.g., baseline physical performance tests such as the Timed Up and Go test, or 6 min walking test) | 5 | 1-6 | 0-10 | 7 | 2-8 | 0-10 | 2 | 0-4.75 | 0-10 |
| Hyperalgesia/Allodynia/Dysesthesia | 4 | 0.5-7.5 | 0-10 | 2 | 0-4.75 | 0-6 | 0 | 0-1.5 | 0-5 |
| Peripheral neuropathy (gross neurological examination) | 4 | 0-6 | 0-10 | 3 | 0-5 | 0-6 | 0 | 0-3.5 | 0-7 |
| Peripheral vascular examination (arterial and venous) | 1 | 0-3.75 | 0-10 | 1 | 0-3.75 | 0-8 | 0 | 0-0 | 0-4 |
| Measures of pain centralization (e.g., dolometric measures) | 5 | 1.5-7 | 0-10 | 2.5 | 0-3.75 | 0-8 | 0 | 0-1 | 0-5 |
|  |  |  |  |  |  |  |  |  |  |
| CRP^§^ | 1.5 | 0-4.5 | 0-8 | 1 | 0-3 | 0-8 | 1.5 | 0-4.5 | 0-10 |
| Erythrocyte sedimentation rate | 1 | 0-3 | 0-8 | 0.5 | 0-3 | 0-7 | 0.5 | 0-3 | 0-10 |
| Serum hyaluronate | 0 | 0-1.75 | 0-8 | 0 | 0-1 | 0-7 | 3 | 0-5 | 0-10 |
| Biomarkers (e.g., CTX-II, COMP) | 1 | 0-3 | 0-8 | 0 | 0-1 | 0-7 | 3 | 0.5-6.5 | 0-10 |
|  |  |  |  |  |  |  |  |  |  |
| Kellgren-Lawrence grading (weight bearing)^§^ | 6 | 2-9 | 0-10 | 6 | 2.5-9 | 0-10 | 9 | 8-10 | 0-10 |
| Bone marrow lesions^§^ | 5 | 3-7 | 0-10 | 5 | 1-6.5 | 0-10 | 8 | 7-10 | 0-10 |
| Joint space width | 5 | 2.5-6 | 0-10 | 5 | 2.5-6.5 | 0-10 | 9 | 8-10 | 0-10 |
| Synovitis (US, MRI)^§^ | 5 | 2-7 | 0-10 | 4 | 2-6.5 | 0-10 | 7 | 5.5-8.5 | 0-10 |
| Effusion (US, MRI)^§^ | 5 | 2-7 | 0-10 | 4 | 2-7 | 0-10 | 7 | 4.5-8 | 0-10 |
| Involved joint compartment (i.e., patellofemoral vs. tibiofemoral vs. combined etc.) | 6 | 3-8 | 0-10 | 6 | 3.5-8 | 0-10 | 8 | 6.5-10 | 0-10 |
| Meniscus tear | 4 | 1-6.5 | 0-10 | 3 | 1-6 | 0-9 | 8 | 4.5-9.5 | 0-10 |
| Meniscus extrusion^§^ | 2 | 0-6 | 0-10 | 2 | 0-5 | 0-8 | 7 | 4-9 | 0-10 |
| Meniscal calcification | 2 | 0-3.5 | 0-10 | 2 | 0-3 | 0-8 | 5 | 3-7 | 0-10 |
| ACL-tear^§^ | 2 | 0-5 | 0-10 | 4 | 0-5 | 0-8 | 7 | 5-8 | 0-10 |
| PCL-tear | 2 | 0-5 | 0-10 | 3 | 0-5 | 0-8 | 6 | 3-7.5 | 0-10 |
| Osteonecrosis | 4 | 0-7.5 | 0-10 | 4 | 0-7 | 0-10 | 6 | 2.5-8.5 | 0-10 |
| Chondrocalcinosis | 2 | 0-5 | 0-10 | 2 | 0-4.5 | 0-10 | 5 | 1-7 | 0-10 |
| Cartilage abnormalities (MRI) | 2 | 0-5 | 0-10 | 2 | 0-4.5 | 0-10 | 7 | 3-9 | 0-10 |
| Malalignment (hip-knee-ankle mechanical axis) | 3 | 1-6.5 | 0-10 | 5 | 2-5.5 | 0-9 | 8 | 5.5-8.5 | 0-10 |
| Cartilage volume/thickness in the compartment of interest (MRI) | 1 | 0-5 | 0-10 | 2 | 0-5 | 0-10 | 8 | 5.5-9 | 0-10 |
| T-1 rho, T2 Mapping of Cartilage | 0 | 0-4 | 0-10 | 0 | 0-4 | 0-10 | 5 | 2-7 | 0-10 |

Legend: Complete list of baseline characteristics and ratings of the importance of each characteristic according to different outcomes, based on opinions of an expert panel

Abbreviations: ^§^ Baseline characteristics listed in the prepared list sent to the experts in the first round of the survey.

**Appendix 2:** Interventions, outcome measures, and results for four fictitious trials presented to the expert panel

|  | Intervention | Outcome | Result |
| --- | --- | --- | --- |
| Study A | Non-surgical treatment versus placebo | Pain | **Significantly** **improved** in the treatment group compared to the placebo group |
| Study B | Non-surgical treatment versus placebo | Pain | **No difference** between the treatment and placebo groups |
|  |  |  |  |
| Study C | Non-surgical treatment versus placebo | Function | **Significantly improved** in the treatment group compared to the placebo group |
| Study D | Non-surgical treatment versus placebo | Function | **No difference** between the treatment and placebo groups |
