# Supplementary material for: Relevant baseline characteristics for describing patients with knee osteoarthritis: results from a Delphi survey
Source: BMC Musculoskelet Disord. 2013 Dec 30;14:369. doi: 10.1186/1471-2474-14-369 (PMC3882493; doi:10.1186/1471-2474-14-369)
Supplement: Additional file 2 — Interventions, outcome measures, and results for four fictitious trials presented to the expert panel. [file 1471-2474-14-369-S2.docx]

Point by point reply

|  | Reviewer’s comment | Our reply |
| --- | --- | --- |
| 1 | Line 35-36: Global knee pain was the only characteristic that achieved a consensus (median ± 2 points) among the experts. | Thank you for this comment: for better understanding we changed the sentence and it reads now: “Global knee pain was the only baseline characteristic that fulfilled among experts the predefined consensus criteria.” The defining criteria are described in the method section of the manuscript. |
| 2 | - Line157-159: The final list only included baseline characteristics with a median rating ?7 (on a 0 to 10 point scale). We arbitrarily defined a consensus among experts as a rating with an interquartile range ?4 (±2) points.  Authors  have to explain why they choose Median instead of mean, and what is the ?interquartile range?. | We prefer the median because outliers or a skewed distribution of values have less impact on the median compared to the mean. Explanation for the meaning of 25-75% interquartile range will follow in comment 3. |
| 3 | Line 191-192: Details of the median, 25-75% interquartile range, and the range of estimates are shown in Appendix 1.  Authors have to explain how the Medianm the IQR, and the Range are calculated. Without those explanations, the readers without the necessary statistical knowledge would not understand. A paragraph in the materials and methods will resolve the problem. | We add explanations for the terms in the materials and method section in paragraph with the subtitle “Statistical analysis”  The median is a measure for the average and the 25-75% Interquartile range (IQR) and the range are measures of dispersion of values. The median value means that half of the values are below and half the values above the median value. The 25-75% IQR is the difference between the values of the 25^th^ and 75^th^ percentiles. The 0^th^ and 100^th^ percentiles (minimal and maximal values) define the range. |
|  | Line 197-200: A consensus on the relevance of a baseline characteristic was arbitrarily defined as a calculated range of four (± 2) points or less around the median. Only one parameter, the global knee pain (e.g., VAS, WOMAC), fulfilled this criterion; all other characteristics displayed ranges greater than four points.  Does this paragraph mean that ?age, gender, BMI, function of knee, duration since onset of symptoms indicating knee osteoarthritis? are no more taken in account?" | Thank for pointing on a passage that could be misunderstood.  In the paragraph beginning with line 198; A consensus on the relevance …. We added at the end of the second sentence… all other characteristics listed in **Table 1** displayed ranges greater than four points but are still rated as relevant baseline characteristics.  We hope that the passage is easier to understand now. |
|  | Acknowledgements ? We strongly encourage you to include an 'Acknowledgements' section between the Authors? contributions section and Reference list. Please acknowledge anyone who contributed towards the study by making substantial contributions to conception, design, acquisition of data, or analysis and interpretation of data, or who was involved in drafting the manuscript or revising it critically for important intellectual content, but who does not meet the criteria for authorship. Please also include their source(s) of funding. Please also acknowledge anyone who contributed materials essential for the study. Authors should obtain permission to acknowledge from all those mentioned in the Acknowledgements.  Please list the source(s) of funding for the study, for each author, and for the manuscript preparation in the acknowledgements section. Authors must describe the role of the funding body, if any, in study design; in the collection, analysis, and interpretation of data; in the writing of the manuscript; and in the decision to submit the manuscript for publication. | Many thanks for the suggestion.  All who contributed to the study are listed as authors. Therefore we have nobody who could be listed in the Acknowledgements section.  We add under rubric competing interests, page 14. The funding bodies had no influence on the design of the study, the analysis of data and the writing of the manuscript. |
